# Illustrating the Value of Critical Methodologies Through Third-sector Gender Studies: A Case for Pluralism

**DOI:** 10.1007/s11266-021-00425-8

**Published:** 2021-11-10

**Authors:** Jennifer Dodge, Angela M. Eikenberry, Tracey M. Coule

**Affiliations:** 1 Rockefeller College of Public Affairs & Policy, University at Albany, SUNY, Albany, USA; 2 School of Public Administration, University of Nebraska at Omaha, Omaha, USA; 3 Sheffield Business School, Sheffield Hallam University, Sheffield, UK

**Keywords:** Critical theory, Critical methodologies, Gender, Third-sector

## Abstract

To encourage methodological pluralism in the field, this paper examines an illustrative sample of articles that apply critical approaches to third-sector studies focused on gender. Specifically, the paper analyzes three articles that were previously identified as among the most critical work on gender in the field between 1970 and 2009 to illustrate how critical research is produced and the value it brings to third-sector studies. We find this work: uncovers hidden assumptions and/or uncomfortable erasures that mask gender-based inequities and injustices; resists hegemonic scientific norms in doing and writing research; and rejects ‘woman’ as a uniform object of theorizing. We discuss against what methodological standards such work should be evaluated and suggest a wider understanding of these ‘alternative’ standards, which might derive significant benefits for the field through increased critical scholarship and the unique features it brings.

## Introduction

This paper examines illustrative examples of how third-sector researchers focused on gender studies apply critical methodologies in their work. Informed by critical epistemologies, critical methodologies foreground such issues as the relationship between knowledge, power, and politics (Foucault, 1969) and thus privilege alternative knowledge assumptions and production processes. As we show, they bring attention to the conditioning effects of social, economic, cultural, and political structures—such as capitalism, patriarchy, or imperialism—on orthodox practices and understandings (Agger, 1998; Keucheyan, 2013; Lee, 1990). With this explicit attention to structure, critical methodologies aim to expose oppression and inequity and analyze pathways toward social change, which requires researching and writing differently from mainstream (positivistic) standards (Ericsson & Kostera, 2020; Gilmore et al., 2019).

We demonstrate that a major contribution of critical approaches is their capacity to unearth the invisible as well as challenge assumptions in the field and society more generally. As a field purporting to be concerned with social betterment, doing research that connects third-sector organizations and organizing to structural issues and institutions is key to bringing about change. Racist ideas and policies, climate change, the COVID-19 pandemic, and economic inequality are intersecting major crises challenging our very existence today. Interrogating the role(s) the third-sector plays in propelling, ameliorating, or abolishing these overlapping challenges is essential. As our gender-based exemplars illustrate, critical research centers the notion of intersectionality and how people with marginalized gender or gender-identities may experience oppression differently than others in these and other societal crises and opens up alternative possibilities and pathways to change. Yet, there is increasing conservatism in critical work in the field (Coule et al., 2020). We discuss this in the conclusion, when drawing out the implications of critical approaches for the field.

Our previous review of critical work published in *Voluntas*, *NVSQ* and *NML* between 1970 and 2009 (Coule et al., 2020) classifies only 4% of all published articles in this period as falling within the ‘critical project’ and those that do are becoming more methodologically ‘mainstream’ over time. In this article, we examine three of the most critical exemplars of third-sector gender studies identified in this earlier research to introduce and illustrate the unique aspects of critical methodologies. We are not suggesting that gender-related studies are the only or most critical third-sector studies. Indeed, our wider review identifies a long tradition of articles that provide similar critiques and display similar methodological features relating to philanthropy (e.g., Fischer, 1995), Global Civil Society (e.g., McDuie-Ra, 2007), nonprofits and changing societies (e.g., Horch, 1994), nonprofits and civic virtue (e.g., Rosenzweig, 1977), and so on.^1^ Rather, we chose this focus because gender-(in)equality is a well-documented social issue, which continues to receive attention from critical third-sector studies scholars (e.g., Korolczuk, 2014; Parente & Martinho, 2018; Phillips, 2015).

Within the space constraints, a full methodological analysis of all the critical work we identified, or all the gender-related studies that have been produced beyond our initial review period (1970–2009), would be impossible. Nor are we able to conduct a full assessment of the pros and cons of critical research vis-à-vis other research traditions in third-sector studies. Our aim is to illustrate how the critical methodologies used in the selected works can help the field to understand often ignored or hidden aspects of the third-sector and the knowledge produced about it in novel ways. We do this to encourage a truly pluralistic view of the field, rather than to suggest that critical scholarship is the only or best approach to research; no single theoretical vocabulary or tradition is capable of a rich and total understanding of organizing within the third-sector.

The rest of the paper is organized as follows. First, we provide a brief overview of critical approaches to research for those who may be less familiar. Next, we provide an assessment of the use of critical methodologies in three third-sector gender studies articles. From this assessment, we discuss recommendations for greater reflection on issues of knowledge production for scholars, editors, and reviewers of critical work in the field. Finally, we discuss implications, considerations, and limitations for scholars doing critical research and for reviewers or editors interested in supporting methodological pluralism.

## Overview of Critical Approaches to Research

There are three general paradigms of research in the social sciences: explanatory or positivist, interpretive, and critical or normative (White & Adams, 1994). These overarching labels encompass work that is significantly and increasingly textured and not always easily delineated. Nevertheless, any form of methodological engagement is founded upon metatheoretical commitments associated with such paradigms, whether implicit or explicit (Coule, 2013; Dodge, 2015). These differ on their views of the nature of reality, purposes for doing research, types of data and methods judged as being valuable and worthwhile, ways of deriving meaning from the data gathered, and the relationships between research and what and who is researched (for a fuller comparison of these different ‘ways of knowing’ see Willis, 2007). Divergent commitments thus have profound implications or consequences for research design, what we take to be ‘data,’ how we collect and analyze data, what we see as (good) ‘theory,’ how we write research accounts, and ultimately the nature of the knowledge claims we make (Cunliffe, 2010).

Critical scholars tend to understand realities to be shaped by (often hidden) social, political, cultural, economic structures and values. Through shifting power dynamics, these structures and values crystallize over time to appear ‘real’ but are understood by critical scholars as changeable (Guba & Lincoln, 2005). Critical researchers typically apply theory as a lens with which to view these phenomena, to see taken-for-granted structures and values in new ways, not to just measure what is there, but to trace connections to theory and social systems (see for example Meyer et al. (2021) for a contemporary assessment of the status of LGBTQ people and Queer Theory in third-sector research and Danley and Blesset [forthcoming] who apply Critical Race Theory to better understand whiteness and segregation in the sector). The theoretical, even ideological, focus of such research can help to uncover hidden assumptions, uncomfortable erasures in society that mask inequities, and injustices that allow inequities to persist.

Critical scholarship also reframes ‘bias’ as ‘perspective’ that serves as an epistemic resource to generate new concepts, methods and questions that open new facets of social and organizational worlds (e.g., Coule and Bain’s, (2021) account of a 3-year embedded ethnography). Critical research thus problematizes dominant conceptions of objectivity. Mainstream (positivist) social science adopts the position that objective methods (i.e., those that purportedly rule out the biases of emotionally engaged “knowers”) enable social scientists to evade errors associated with their projection of value-judgments onto the phenomena and/or population of interest and reach an absolute understanding of how things ‘really’ are. Achieving this objectivity often rests upon emotional *detachment* of the knower from the known, *value-neutrality* in the evaluation of the known, observation of the regularities of an object under (experimental or statistical) *control,* and a *subject-object dichotomy* where what is real is taken to exist independently of the knower. Most of the studies that took gender as their primary focus in our original review (Authors, 20XX) remained closer to these standards than the three we discuss below (e.g., Gibelman, 2000; Sampson & Moore, 2008).

Foundational to all critical methodologies is the notion that research derives much of its analytical power from meta-theory. Therefore, it is not possible to separate considerations of methodology from considerations of theory. In addition, while critical methodologies might rely on methods used by researchers influenced by other research paradigms, their use is informed by very different knowledge constituting assumptions, purposes, and processes (Coule, 2013). That is, just because a researcher uses qualitative methods does not necessarily mean they do so in the spirit of critical inquiry. Further, the view that quantitative methods belong to the positivistic paradigm and qualitative methods belong to an interpretive or critical paradigm is too simplistic (see Duberley et al., (2012) on ‘qualitative positivism’ and Thorne, (1997) on ‘naïve empiricism’).^2^

In this paper, we focus specifically on critical gender studies, which are concerned with how gender does and ought to affect ideas about knowledge, knowers (and their relationship to the known), methodological practices, and justification. Central to this is an analysis of the ways in which women, queer, or gender minorities, and their intersectionality with other marginalized groups, are disadvantaged by dominant ideas and practices, alongside efforts to reform them in the interests of such groups.

## Assessment of Critical Third-sector Studies Research on Gender

The three exemplar studies discussed below represent the most (radically) critical of the fourteen gender-related studies in our original review (Coule et al., 2020), insofar as they embody multiple tenets of critical research as outlined by Adler et al., (2008): challenging structures of domination, questioning taken-for-granted assumptions, going beyond instrumentalism, and paying attention to power and knowledge. These studies deal with various aspects of the third-sector, including volunteering, social services for battered women, and feminist methodology, to reveal gendered work practices in third-sector organizing and research. They also draw explicitly on feminist theoretical resources to advance their analyses, with two also taking feminist organizing as their “object” of study. See Table 1 for an overview of the three articles. The main themes drawn from this work are that they: uncover hidden assumptions and/or uncomfortable erasures that mask gender-based inequities and injustices; resist hegemonic scientific norms in doing and writing research; and reject ‘woman’ as a uniform object of theorizing.

**Table 1:** Gender-focused critical third-sector studies articles

Author(s)	Year	Journal	Title	Topic	Methodology	Method(s)	Data Source(s)
Christiansen-Ruffman, L	(1985)	NVSQ^1^	Participation Theory and the Methodological Construction of Invisible Women: Feminism's Call for Appropriate Methodology	Research Methodology	Literature Review	Content Analysis	All volumes of the *Journal of Voluntary Action Research*, 1972–1983
Metzendorf, D. & Cnaan, R. A	(1992)	NML^2^	Volunteers in Feminist Organizations	Volunteering; Feminist Organizations	Multiple Case Study	Interviews, Document Analysis	15 feminist organizations in the Delaware Valley, Pennsylvania
Kenney, S. J	(2005)	NML	Domestic Violence Intervention Program: Unconditional Shelter?	Services to Battered Women	Single Case Study	Not discussed	Not discussed

^1^Nonprofit & Voluntary Sector Quarterly

^2^Nonprofit Management and Leadership

### Uncovering Hidden Assumptions and/or Uncomfortable Erasures That Mask Inequities and Injustices

In different ways, these articles uncover hidden assumptions and erasures in third-sector practices and knowledge production processes. In her 1985 article, Christiansen-Ruffman provides a thorough literature-based analysis of how dominant knowledge practices in the field disadvantage women by rendering them and their work in the academy and in the sector invisible. She points to their exclusion from inquiry, denial of their epistemic authority, and the production of theories that render invisible their activities and interests or gendered power relations, or that reproduce gender hierarchies. She traces such failures to flawed notions of scientific knowledge and methodology and offers suggestions for how to overcome them.

Metzendorf and Cnaan, (1992) critique societal expectations of women volunteers, highlighting how volunteering can be a form of exploitation and challenging the assumption that feminist organizations and their management practices exemplify the ideology they exist to advance in society, namely women’s equality. Through their analysis of interviews and documents across multiple case studies, they trace the erosion of feminist principles and the adoption of utilitarian ones over time in these organizations. They found that volunteers were increasingly viewed as resources rather than colleagues and took on increasingly menial tasks rather than participating in decision making or other leadership activities.

Kenney, (2005), through a case study of a domestic violence shelter in crisis, highlights how class, sexual orientation, race, and feminist ideology structure nonprofit work, in this case services to battered women. It also provides a window into the difficult legal issues at play in protecting (or not) women who have experienced intimate violence.

By foregrounding the ways gender fundamentally organizes society (Hawksworth, 2010), these articles offer fresh interpretations of third-sector organization behavior. This approach corrects for bias in previous third-sector research, either by centering women’s experiences in organizations, which were previously invisible, or viewing third-sector organizations from a gendered lens to show how gender organizes social and organizational relations and processes.

### Resisting Hegemonic Scientific Norms of Doing and Writing Research

The three articles show how critical scholarship contravenes scientific norms in several ways. In terms of ‘doing’ empirical research, these scholars privilege qualitative methodological practices that hold the potential to avoid replication of power differences between the knower and the known. Metzendorf and Cnaan and Kenney appear to have spent significant time in the field, using inquiry methods that seek out and accept women’s accounts of their experiences in situ and in their own terms. In other words, inquiry itself is treated as a social and socially located practice, generating knowledge that is contextual, relational, embodied, and dialogical (Cunliffe, 2018).

In writing her research account, Christiansen-Ruffman constructs herself as an active, situated agent in the research process by writing in the first person and making explicit reference to personal experience in relation to the subject matter. Kenney does not adhere to structural conventions of mainstream scientific writing, providing no reference to or justification for the methodological practices underpinning her case study.Yet the account provided leaves no doubt as to the rich and extensive nature of the data underpinning the paper. Kenney adopts a storytelling style from the outset, opening the article with a vivid depiction of Beth George (a central character in the story) and the shelter where she resided and subsequently worked. In this way, she provides a thoroughly humanizing account of both organizational life and the life of individual research participants (i.e., “the known”).

There is, however, a paradox in the situatedness of these accounts. While the articles have used “engaged” methods of inquiry in the field to situate research participants and consider how their social location (identity, relations, roles, role-given interests) affects their social realities, experiences and (in)visibility, the authors’ own situatedness or positionality as the ‘knower’ is rendered invisible. By writing from the vantage point of third person and remaining silent on how their own location affects (representations of) how and what they know, Kenney and Metzendorf and Cnaan attempt to stand outside their own written artifacts. In the act of writing, they privilege their own epistemic authority in constructing their representations and do not represent women in their own terms and words, which were no doubt observed in the field. In this sense, the methods of inquiry and the methods of writing seem at odds with one another. Metzendorf and Cnaan’s paper conforms most closely to mainstream (positivist) scientific norms regarding structure, style, and content. It contains a conventional methods section that concerns itself with procedure, sampling, data collection and analysis. The language is disembodied with the authors referring to: themselves as “the researchers”; archival data being made available “for this study”; and “using” 15 organizations as the sample. Significant emphasis is placed on stressing randomness and representation within the sample and alleviating concerns about the wide variation in size, budget, mission, and location as ‘none of these variables was significantly associated with issues of volunteer use’ (p. 261).

### Rejecting ‘Woman’ As a Uniform Object of Theorizing

Two of the articles adopt an intersectional view of gender identity. Kenney’s case study examines how class, sexual orientation, and race intersect with feminist ideology to structure services to battered women. Powerfully, she calls on the reader to critically reflect on how these complex identity politics might influence how we decide what and whom to believe. Metzendorf and Cnaan consider the role of social class in gendering volunteering. Addressing these issues attempts to mitigate the reproduction and reinforcement of asymmetrical power relations that would arise from assuming white, middle class heterosexual women can and should define and speak for the standpoint of *all* women. Instead, these studies interrogate and expose the exclusionary tendencies within feminism and feminist organizing itself, thus providing a robust reflexive “internal critique” of the very theoretical resources they deploy.

These themes could be applied to a range of issues to open up new areas of scholarship. For instance, Christiansen-Ruffian shows how sexist biases in citizen participation research can lead to a failure to recognize the fundamental nature and implications of gender in society. One could examine the extent to which practices related to collaboration might reflect gendered assumptions and how this shapes power and authority in these relationships. One might also examine board governance and how intersecting identities might manifest in decision making processes or leadership practices. There are many possibilities for generating new insights, and we encourage readers to reflect on how the principles of critical inquiry could positively influence their own research questions, processes, and outcomes.

## Evaluating Critical Methodologies

Our assessment above suggests the need for different standards than mainstream positivist-oriented ones to evaluate critical research to support greater plurality in the field. Without a widespread understanding of these different standards, the field risks the ongoing dampening and subordination of critical scholarship and the unique benefits it brings, not only in terms of offering new interpretations of phenomenon of central interest to the field, but also in raising moral and ethical considerations in knowledge production processes generally.

As the exemplars illustrate, critical researchers aim to generate insights that challenge taken-for-granted assumptions and ultimately change our basic understanding of some phenomenon, thereby contributing in specific ways to knowledge production in the field. Thus, evaluation of this work might consider whether an article provides an interpretation of a phenomenon that challenges basic assumptions—in scholarship or third-sector organization practice—and convincingly reveals some form of oppression or injustice, such as Metzendorf and Cnaan did by showing women’s volunteering in feminist organizations as exploitation rather than liberation. Such scholarship supports “a raised level of awareness” of oppression and increased capacity for moral critique of it, among researchers, research participants, and others (Guba & Lincoln, 2005, p. 207). In critical feminist research, such as that reviewed above, this often takes the form of “deconstruction of the patriarchal forms of oppression in social structures” (Guba & Lincoln, 2005, p. 201), reflecting, again, another key tenet of critical scholarship: to challenge structures of domination.

Critical researchers also attempt to reflexively interrogate “the epistemological and political baggage they bring with them” as well as “how hegemonic regimes of truth affect the subjectivities of the disadvantaged” (Johnson et al., 2006, p. 142). As we discuss above, the authors of the exemplars uphold the critical tenet of foregrounding the relationship between power and knowledge with respect to the topic of study—volunteering or providing services—and engage methods that privilege participants’ subjectivities, against the grain of hegemonic forms of inquiry. However, some authors (Christiansen-Ruffman is the exception) also implicitly eschew that the knowledge claims they construct reflect their particular perspectives as the knower (as well as the known). By using their socially positioned power as researchers to represent participants’ worlds and presenting a façade of their own a-perspectivity or “voice from nowhere” (Lather cited in Guba & Lincoln, 2005, p. 209), they (perhaps inadvertently) objectify and subordinate participants and reproduce the very power asymmetries they oppose, which runs counter to a critical intent.

While the “gold standard” for explanatory or positivist research may be internal validity (whether the study is replicable and the data reliable) and generalizability (looking for universal truths), critical research focuses on “the correctness or credibility of a description, conclusion, explanation, interpretation or other sort of account” (Maxwell, 2013, p. 122). Quality, then, is not found in (the elaboration of) methodological procedures per se but in the giving of accounts and what Guba and Lincoln, (2005) call “defensible reasoning… in ascribing salience to one interpretation over another” (p. 205); that is, *interpretive rigor*. As noted above, the selected articles often reject methodological conventions, and instead of providing extensive executional details they offer rich descriptions and demonstrate their claims through ‘humanizing’ accounts that offer fidelity to (organizational) life as experienced by those who live it. Critical scholars acknowledge that (their) research can only ever offer partial accounts, from particular perspectives, that are open to challenge and revision. While critical scholars are comfortable with this ambiguity, it runs counter to dominant notions of ‘good’ or ‘real’ science within the field.

Responding to a common criticism of critical scholarship—that it illuminates problems but fails to provide solutions—an evaluation of critical work can also assess whether the research suggests and stimulates possible actions to create emancipation, social transformation, equity, and/or social justice (Guba & Lincoln, 2005; Johnson et al., 2006). Linking this criterion (‘action stimulus’) to credibility and trustworthiness, Guba and Lincoln, (2005, p. 205) argue that one should ask, “are these findings sufficiently authentic (isomorphic to some reality, trustworthy, related to the way others construct their social worlds) that I may trust myself in acting on their implications?”. More specifically, does research support “creating the capacity in research participants for positive social change and forms of emancipatory community action?” (Guba & Lincoln, 2005, p. 207). While we would not expect anyone to act on the basis of one account, the papers do suggest possible pathways to action. Christiansen-Ruffman makes a strong case for alternative knowledge constituting assumptions with direct relevance for shaping research practice. Metzendorf and Cnaan and Kenney also raise ‘big questions’ that leaders and other participants in feminist organizations should feel compelled to reflect on in ensuring they implement fair and inclusive practices.

## Critical Research and Methodological Pluralism

Application of critical epistemologies in research has implications for scholars, reviewers and editors interested in supporting methodological pluralism. First, while critical scholars tend to be (more) explicit about the ways knowledge production processes involve moral, social, and political judgments, critical scholarship suggests the importance of raising these questions for the field more generally to recuperate the transformative aspirations of the field, and to bring about positive social change. Mainstream science holds that ‘good’ science or warranted knowledge is neutral in relation to social, moral, and political values. In other words, it is non-normative. Yet, to claim that mainstream (positivistic) theories are merely a reflection of how things really are limits the ability and responsibility of researchers to hold the values that have shaped their inquiry up for critical scrutiny (as, for example, Kenney and Metzendorf and Cnaan do). This is itself a stance of social power.

Second, precisely because critical epistemologies and methodologies exist to advance the cause, status, and interest of subordinated groups and issues, they are often framed in the mainstream as inherently ‘biased’ and as such, ‘bad science’. If researchers, reviewers, and editors continue to assign epistemic authority to positivistic approaches, laying down its set of principles as the incontrovertibly true means to achieve warranted knowledge, critical scholarship will be further stigmatized and subordinated. Such practices surmount to epistemic injustices against researchers in the field who adopt ‘alternative’ knowledge constituting assumptions and inquiry practices. Ultimately, this undermines their ability to contribute to rich understandings of the field, publish the outcomes of their research, and progress in the academy. True acceptance of and reform toward methodological pluralism would protect third-sector studies from further loss of a revolutionary horizon that was once a central part of the field as it emerged in the 1970s (Coule et al., 2020).

One of the implications of the above assessment is that knowledge production processes in academia might often eschew critical research because it is emergent, ‘dirty’ and disruptive not only to patriarchy or other oppressive forces in society, but also to more masculine notions of ‘rigorous’ scientific method (e.g., consider the ‘big data’ movement). Despite the potential benefits of critical scholarship, we and other scholars working in these traditions face myriad pressures to conform to dominant conceptions of ‘good research’ out of step with critical research traditions. Each of us have ‘normalized’ our research accounts to publish in the leading outlets of the field, including shortening our detailed and contextualized accounts of the phenomena we have studied to elaborate on research procedures to satisfy reviewers, a move that goes against the grain of critical notions of quality. We have also ‘mainstreamed’ our accounts of the research process, at least in the write-up if not in the application of inquiry methods themselves, as exhibited in the articles reviewed above. We speculate that this is one of the reasons why critical scholarship is relatively marginal in the field: because reviewers apply positivist criteria to critical work, while critical scholars, including ourselves, must either submit to these criteria in research reporting, or spend considerable effort discussing criteria with editors and reviewers to explain our ‘alternative’ assumptions. This kind of preference for positivist scholarship may have its roots in doctoral training where students can be encouraged to avoid critical scholarship for the sake of their careers and limited time in the classroom is dedicated to understanding the logic of critical scholarship, including evaluating it appropriately.

For these reasons, more attention needs to be given to appropriate criteria for assessing critical research, which are often poorly understood. We thus end this paper with a call for the field to move beyond the universal application of quality criteria appropriate to positivism, including ‘qualitative positivism’, and be more attuned to specific criteria for critical and interpretive research (Duberley et al., 2012) in the peer-review process and in doctoral training. There is also opportunity to discuss further the challenges and possibilities of quantitative critical methodologies and mixed methodologies that might open new horizons of inquiry.

When critical researchers are courageous enough to articulate, and editors and reviewers better understand and accept, the knowledge constituting assumptions and methodologies of critical research and quality criteria by which it should be judged, third-sector studies will more readily benefit from the insights of critical scholarship.

## Footnotes

1 See Coule et al. (2020) for a fuller discussion of third-sector studies in the critical tradition and their evolution.

2 The possibility of mixing paradigms, as some self-described pragmatists advocate, may also be challenging if one subscribes to the premise that positivist epistemology is incommensurate with critical epistemologies.
